# EVIDENCE-BASED REVIEWS & RECOMMENDATIONS FROM THE BRAZILIAN SOCIETY OF SHOULDER AND ELBOW SURGERY: DOUBLE-ROW REPAIR FOR LARGE ROTATOR CUFF TEARS

**DOI:** 10.1590/1413-785220263401e303241

**Published:** 2026-08-21

**Authors:** Eduardo Angeli Malavolta, Leonardo Zanesco, Gustavo de Mello Ribeiro Pinto, Marcus Vinícius Galvão Amaral, Thiago Barbosa Caixeta, Jair Simmer, Luciana Andrade da Silva, Marcelo Costa de Oliveira Campos

**Affiliations:** 1 Universidade de Sao Paulo, Faculdade de Medicina, Hospital das Clinicas HCFMUSP, Sao Paulo, SP, Brazil.; 2 Hcor, Sao Paulo, Brazil.; 3 Universidade do Estado do Rio de Janeiro (UERJ), Rio de Janeiro, RJ, Brazil.; 4 Instituto Nacional de Traumatologia e Ortopedia Jamil Haddad, Centro de Cirurgia do Ombro e Cotovelo, Rio de Janeiro, RJ, Brazil.; 5 Universidade Federal de Goias, Goiania, Goias, GO, Brazil.; 6 Hospital Estadual de Urgencia e Emergencia, Departamento de Ortopedia e Traumatologia, Vitoria, ES, Brazil.; 7 Santa Casa de Misericordia de Sao Paulo, Faculdade de Ciencias Medicas, Departamento de Ortopedia e Traumatologia, Grupo de Cirurgia do Ombro e Cotovelo, Sao Paulo, SP, Brazil.

**Keywords:** Rotator Cuff Injuries, Arthroscopy, Suture Techniques, Shoulder Joint, Treatment Outcome, Evidence-Based Practice, Lesões do Manguito Rotador, Artroscopia, Técnicas de Sutura, Articulação do Ombro, Resultado do Tratamento, Prática Clínica Baseada em Evidência

## Abstract

**Introduction:**

Large (≥ 3 cm) rotator cuff tears have a poorer prognosis, and the optimal arthroscopic repair configuration (single-row vs double-row) remains controversial.

**Methods:**

We performed an evidence-based review of systematic reviews and randomized controlled trials comparing arthroscopic double-row and single-row repair for tears ≥ 3 cm. PubMed, Embase, the Cochrane Library, and BVS were searched through May 2025. Only studies reporting size-stratified outcomes were included. Methodological quality was assessed, and the certainty of evidence was graded using the GRADE approach.

**Results:**

Two high-quality systematic reviews and five randomized trials provided separate data for tears ≥ 3 cm. Double-row repair showed better functional outcomes than single-row repair, with improved ASES (MD −3.09; 95% CI −6.19 to 0.02) and UCLA scores (MD −1.47; 95% CI −2.21 to −0.72) (moderate-quality evidence). Limited randomized data suggested higher healing rates with double-row repair (OR 3.44; 95% CI 1.09–10.88; low-quality evidence). Pain alone did not differ meaningfully beyond the immediate postoperative period.

**Conclusions:**

For rotator cuff tears ≥ 3 cm, arthroscopic double-row repair provides superior functional outcomes and likely higher structural healing versus single-row repair, supporting its preferential use in large and massive tears, with decisions individualized to patient and lesion characteristics. ***Level of evidence II; Systematic review.***

## RESUMO

**Introdução:**

As roturas do manguito rotador de grande tamanho (≥ 3 cm) apresentam pior prognóstico, e a configuração ideal de reparo artroscópico (fileira simples vs fileira dupla) permanece controversa.

**Métodos:**

Realizou-se uma revisão baseada em evidências de revisões sistemáticas e ensaios clínicos randomizados que compararam o reparo artroscópico em fileira dupla e em fileira simples para roturas ≥ 3 cm. As bases de dados PubMed, Embase, Cochrane Library e BVS foram pesquisadas até maio de 2025. Foram incluídos apenas estudos que relataram desfechos estratificados pelo tamanho da lesão. A qualidade metodológica foi avaliada, e a certeza da evidência foi graduada segundo a abordagem GRADE.

**Resultados:**

Duas revisões sistemáticas de alta qualidade e cinco ensaios randomizados forneceram dados específicos sobre roturas ≥ 3 cm. O reparo em fileira dupla apresentou melhores desfechos funcionais do que o reparo em fileira simples, com escores superiores de ASES (DM −3,09; IC95% −6,19 a 0,02) e UCLA (DM −1,47; IC95% −2,21 a −0,72) (evidência de qualidade moderada). Dados randomizados limitados sugerem maior taxa de cicatrização com a fileira dupla (OR 3,44; IC95% 1,09–10,88; evidência de baixa qualidade). A dor, isoladamente, não diferiu de forma clinicamente relevante após o período pós-operatório imediato.

**Conclusão:**

Para roturas do manguito rotador ≥ 3 cm, o reparo artroscópico em fileira dupla oferece melhores desfechos funcionais e provavelmente maior cicatrização estrutural em comparação ao reparo em fileira simples, o que apoia sua preferência em lesões grandes e maciças, com decisões individualizadas às características do paciente e da própria lesão. ***Nível de Evidência II; Revisão sistemática.***

## RECOMMENDATION

There is moderate-quality evidence demonstrating that double-row repair of rotator cuff tears ≥ 3 cm is superior to single-row repair in terms of shoulder function and pain, as assessed by the ASES and UCLA shoulder scales (Level of Evidence I). There is low-quality evidence indicating that the healing rate of the rotator cuff after surgery is higher among patients who underwent double-row repair (Level of Evidence I).

The SBCOC Consensus Project recommends that surgeons should individually assess the patient- and lesion-specific characteristics to make the best treatment decision. According to current evidence, double-row rotator cuff repair provides superior clinical and structural outcomes in large and massive tears (≥ 3 cm) and should therefore be preferred in these cases.

## INTRODUCTION

Rotator cuff tears are the most common cause of shoulder disability and are possibly the most frequent tendon disorder in the human body. The prevalence of this condition has increased proportionally with the aging of the population.^1^

Tear size is commonly classified in the literature as small, medium, large, or massive (small: <1 cm; medium: 1–3 cm; large: 3–5 cm; massive: >5 cm). Massive tears generally involve multiple tendons.^2^ Arthroscopic single-row rotator cuff repair has traditionally been considered the standard technique, although several clinical studies have reported significant retear rates following this approach.^3^ Recent studies have shown that double-row repair can improve the anatomical footprint restoration, increase tendon strength, expand the acromiohumeral distance, enhance postoperative function, and reduce retear rates.^4^ On the other hand, the double-row technique is technically more demanding, involves a longer surgical time, and consequently increases the cost of the procedure compared with single-row repair. Nevertheless, the cost-effectiveness ratio remains favorable for the double-row technique.^5^

The optimal technique for rotator cuff repair remains controversial in the literature, as many studies have not found substantial differences in clinical outcomes when comparing single- and double-row repairs. However, it is essential to assess outcomes according to tear size, since large and massive tears are associated with worse prognoses.^6^

This evidence review was developed within a collaborative initiative between Cochrane and the Brazilian Society of Shoulder and Elbow Surgery (SBCOC). The aim of this partnership is to synthesize the best available evidence using rigorous, transparent methods and to translate it into clear, practical recommendations for clinical practice.

### Research Question

Is double-row repair more effective than single-row repair for rotator cuff tears ≥ 3 cm?

## METHODS

- Databases searched: PubMed, Embase, Cochrane Library, and BVS.
- Descriptors: "Rotator Cuff Injuries" [Mesh]; "Arthroscopy" [Mesh]; "single row"; "double row" or "dual-row"; Systematic Review [Publication Type]; Randomized Controlled Trial [Publication Type] (see Appendix 1).
- Search period: Up to May 2025.
- Included studies: Systematic reviews (SRs) of the literature with good methodological quality and up-to-date evidence were prioritized. In the absence of systematic reviews, randomized controlled trials (RCTs) with adequate methodological quality were also considered.

## RESULTS

The search identified 98 references (61 in PubMed, 25 in Embase, and 12 in the Cochrane Library). Among these, 22 were systematic reviews^4, 7–27^ — the prioritized study type for this assessment — and 15 were randomized controlled trials (RCTs).^28–42^

**Figure 1:**
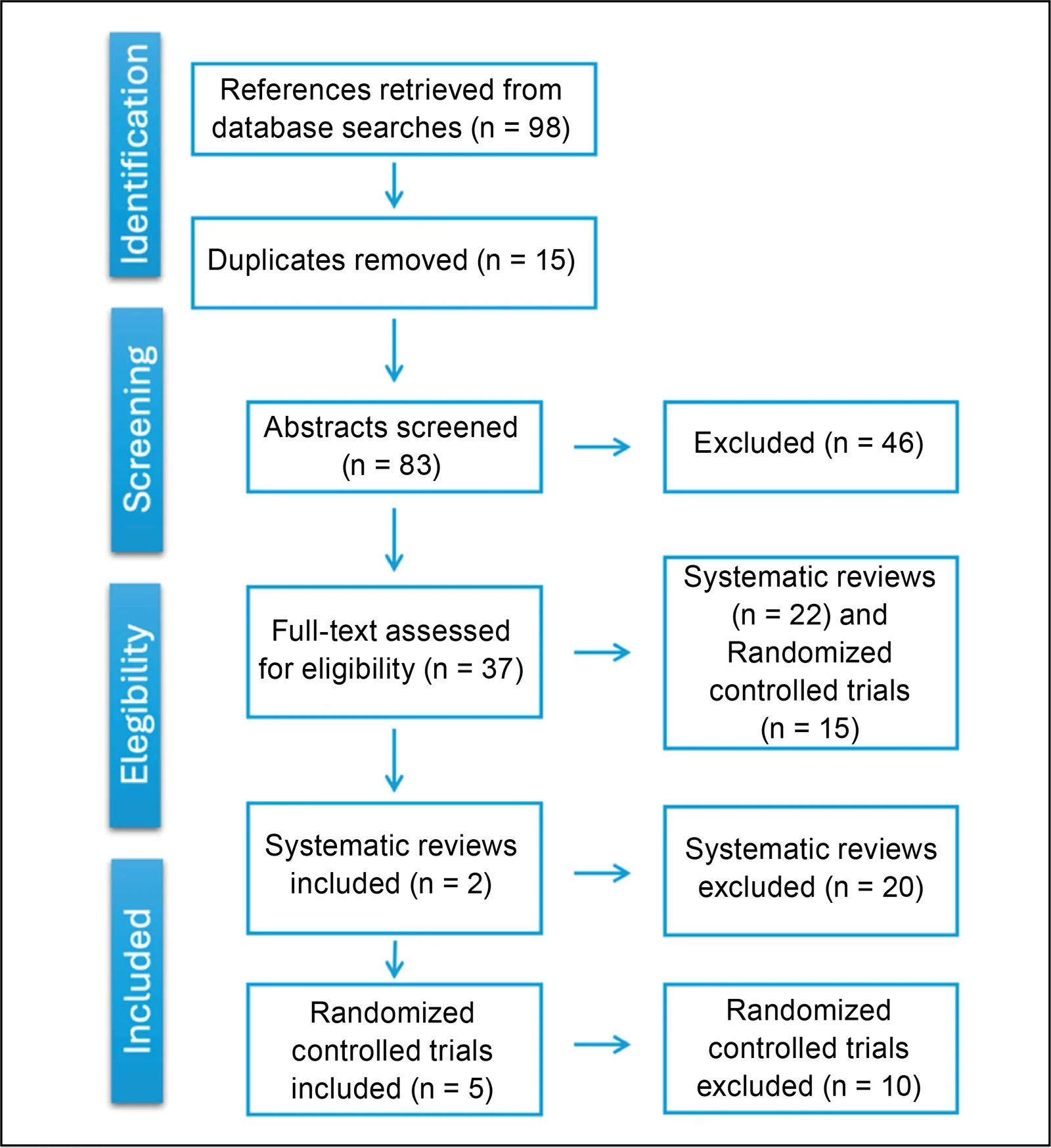
Flow diagram of the literature search.

After screening the systematic reviews, 20 were excluded for including rotator cuff tears smaller than 3 cm without stratification by tear size. Among the 16 RCTs, only five presented separate results for tears ≥ 3.0 cm.^28, 30, 31, 34, 38^ (Figure 1).

### Included studies

Of the 22 systematic reviews identified, only two analyzed tears ≥ 3 cm separately.^7–12^ The most recent review assessing outcomes by tear size was published by Gu et al. (2023) [12]. This systematic review with meta-analysis compared clinical outcomes after single-row and double-row rotator cuff repair for tears of different sizes. The methodological quality was assessed using the Joanna Briggs Institute (JBI) critical appraisal tool for systematic reviews and evidence syntheses,^43^ concluding that the review had good methodological quality.

The review included 10 controlled studies (7 RCTs, 1 prospective cohort, and 2 retrospective studies), comprising 404 double-row and 387 single-row repairs in total. The mean follow-up was 55.1 months (range 24–168), and the included studies were judged to be of high quality by the authors. For functional outcomes, shoulder pain, and disability measured by the American Shoulder and Elbow Surgeons (ASES) score, double-row repair for tears ≥ 3 cm yielded better ASES scores than single-row repair: mean difference (MD) = −3.09 (95% CI: −6.19 to 0.02; p = 0.05; 3 studies; 99 patients). Using the University of California Los Angeles (UCLA) shoulder scale, which assesses pain, function, and patient satisfaction, a significant difference favored the double-row technique over single-row: MD = −1.47 (95% CI: −2.21 to −0.72; p < 0.01; 4 studies; 143 patients).

For the retear outcome, the review identified only one randomized study, in which significantly more retear events occurred among patients who underwent single-row repair (6/12) compared with those who underwent double-row repair (1/14) (OR = 13.00 [95% CI, 1.27 to 133.28]; P = .03). The only review that specifically evaluated the healing rate after rotator cuff repair was conducted by Chen et al (2013).^7^ This systematic review included 12 studies, of which six were RCTs. The authors found that participants who underwent double-row repair demonstrated higher healing rates compared with those who received single-row repair (OR = 3.44; 95% CI: 1.09–10.88; p = 0.035; 3 studies; 87 patients). No additional randomized studies assessing this outcome were identified after the publication of this review.

Pain is one of the domains assessed by the UCLA shoulder scale and the ASES instruments; therefore, few studies analyzed this outcome separately. In the review by Gu et al.^12^, among the included randomized trials, only two reported specific results for pain. Imam et al.^34^ observed a significant early difference 24 hours after surgery in VAS pain scores between the single-row and double-row groups (p < 0.01), favoring single-row repair. However, at six weeks postoperatively, this difference was no longer observed. Li et al.^37^ reported that both groups showed postoperative pain improvement with no significant difference between them (p = 0.56).

The general characteristics of the evaluated studies and the quality of evidence according to the GRADE system^44, 45^ are presented in Tables 1 and 2, respectively.

**Table 1:** Characteristics of the included studies.

Included Studies	Type of Study	Studies Included (Study Design)	Sample Size	Previously Published Protocol
Gu et al (2023)	Systematic review	10 (7 RCTs, 3 cohorts)	791	No
Krogh et al. (2012)	Systematic review	12 (6 RCTs, 6 cohorts)	476*	No

RCTs :randomized clinical trials;

* reported only from randomized trials.

**Table 2:** Quality of evidence (GRADE).

Outcome	Studies (N)	Relative effect (95% CI)	Quality of evidence
Function, Pain, Disability - ASES	3 studies (99)	DM −3.09 (IC 95%: −6.19 a 0.02)	Moderate^1^
Function, Pain, and Satisfaction - UCLA	4 studies (143)	DM= −1.47 (IC 95%: −2.21 a −0.72)	Moderate^1^
Healing	3 studies (87)	OR= 3.44 (IC 95%: 1.09 a 10.88)	Baixa^1^,^3^

1 Limited sample size

2 Imprecision (large confidence interval)

3 Bias risk of the included studies. Note: GRADE (Grading of Recommendations Assessment, Development, and Evaluation) is a tool used to assess and measure the quality of evidence. It is based on five pillars: Risk of Bias, Imprecision, Inconsistency, Indirect Assessment of Outcomes, and Publication Bias. Thus, the quality of evidence is classified as very low, low, moderate, or high quality^44,45^.

## CONCLUSION

There is moderate-quality evidence indicating that double-row repair of rotator cuff tears ≥ 3 cm is superior to single-row repair in terms of function and pain, as assessed by the ASES and UCLA shoulder scales. There is low-quality evidence suggesting that the healing rate after surgery is higher in patients who underwent double-row repair.

## Data Availability

The data underlying the research text are contained in the manuscript.

## Appendix 1 Database search strategies.

**Table:**

**PUBMED**
#1 "Rotator Cuff Injuries"[Mesh] OR (Cuff Injury, Rotator) OR (Injury, Rotator Cuff) OR (Rotator Cuff Injury) OR (Rotator Cuff Tears) OR (Rotator Cuff Tear) OR (Tear, Rotator Cuff) OR (Tears, Rotator Cuff) OR (Rotator Cuff Tendinitis) OR (Rotator Cuff Tendinitides) OR (Tendinitis, Rotator Cuff) OR (Rotator Cuff Tendinosis) OR (Rotator Cuff Tendinoses) OR (Tendinoses, Rotator Cuff) OR (Tendinosis, Rotator Cuff) OR (Glenoid Labral Tears) OR (Glenoid Labral Tear) OR (Labral Tear, Glenoid) OR (Labral Tears, Glenoid) OR (Tear, Glenoid Labral)
#2 "Arthroscopy"[Mesh] OR (Arthroscopies) OR (Arthroscopic Surgical Procedures) OR (Arthroscopic Surgical Procedure) OR (Procedure, Arthroscopic Surgical) OR (Procedures, Arthroscopic Surgical) OR (Surgical Procedure, Arthroscopic) OR (Arthroscopic Surgery) OR (Arthroscopic Surgeries) OR (Surgeries, Arthroscopic) OR (Surgery, Arthroscopic) OR (Surgical Procedures, Arthroscopic)
#3 ("single row" AND "double row")
#4 #4 #1 AND #2 AND #3
Filters applied: Clinical Trial; Meta-Analysis; Randomized Controlled Trial; Review; Systematic Review = 61

**Table:**

**COCHRANE**
#1 "Rotator Cuff Injuries" OR (Cuff Injury, Rotator) OR (Injury, Rotator Cuff) OR (Rotator Cuff Injury) OR (Rotator Cuff Tears) OR (Rotator Cuff Tear) OR (Tear, Rotator Cuff) OR (Tears, Rotator Cuff) OR (Rotator Cuff Tendinitis) OR (Rotator Cuff Tendinitides) OR (Tendinitis, Rotator Cuff) OR (Rotator Cuff Tendinosis) OR (Rotator Cuff Tendinoses) OR (Tendinoses, Rotator Cuff) OR (Tendinosis, Rotator Cuff) OR (Glenoid Labral Tears) OR (Glenoid Labral Tear) OR (Labral Tear, Glenoid) OR (Labral Tears, Glenoid) OR (Tear, Glenoid Labral)
#2 "Arthroscopy" OR (Arthroscopies) OR (Arthroscopic Surgical Procedures) OR (Arthroscopic Surgical Procedure) OR (Procedure, Arthroscopic Surgical) OR (Procedures, Arthroscopic Surgical) OR (Surgical Procedure, Arthroscopic) OR (Arthroscopic Surgery) OR (Arthroscopic Surgeries) OR (Surgeries, Arthroscopic) OR (Surgery, Arthroscopic) OR (Surgical Procedures, Arthroscopic)
#3 ("single row" AND "double row")
#4 #1 AND #2 AND #3 = 12

**Table:**

**EMBASE**
#1 ‘rotator cuff injury’/exp OR ‘cuff injury’ OR ‘cuff lesion’ OR ‘rotator cuff disease’ OR ‘rotator cuff disorder’ OR ‘rotator cuff disorders’ OR ‘rotator cuff injuries’ OR ‘rotator cuff injury’ OR ‘rotator cuff lesion’ OR ‘rotator cuff lesions’ OR ‘rotator cuff pathology’ OR ‘rotator cuff tendinopathies’ OR ‘rotator cuff tendinopathy’ OR ‘shoulder rotator cuff degeneration’ OR ‘shoulder rotator cuff lesion’
#2 ‘arthroscopy’/exp OR ‘arthro-endoscopy’ OR ‘arthroendoscopy’ OR ‘arthroscopic exam’ OR ‘arthroscopic examination’ OR ‘arthroscopic procedure’ OR ‘arthroscopic procedures’ OR ‘arthroscopy’
#3 ("single row" AND "double row")
#4 #1 AND #2 AND #3 = 189
#4 AND ([embase]/lim NOT ([embase]/lim AND [medline]/lim) OR ([medline]/lim NOT ([embase]/lim AND [medline]/lim) NOT ([embase classic]/lim AND [medline]/lim))) AND ([cochrane review]/lim OR [systematic review]/lim OR [meta analysis]/lim OR [controlled clinical trial]/lim OR [randomized controlled trial]/lim) = 25

**Table:**

**PORTAL REGIONAL BVS**
#1 Mh:"Lesões do Manguito Rotador" OR (Lesões do Manguito Rotador) OR (Lesões do Ligamento Glenoidal) OR (Lesões do Ligamento Glenoídeo) OR (Lesões do Ligamento Glenóideo) OR (Roturas da Coifa dos Rotadores) OR (Roturas do Ligamento Glenoidal) OR (Roturas do Ligamento Glenoídeo) OR (Roturas do Ligamento Glenóideo) OR (Roturas do Manguito Rotador) OR (Ruptura do Manguito Rotador) OR (Rupturas da Coifa dos Rotadores) OR (Rupturas do Ligamento Glenoidal) OR (Rupturas do Ligamento Glenoídeo) OR (Rupturas do Ligamento Glenóideo) OR (Rupturas do Manguito Rotador) OR (Tendinite da Coifa dos Rotadores) OR (Tendinite do Manguito Rotador) OR (Tendinose da Coifa dos Rotadores) OR (Tendinose do Manguito Rotador) OR MH:C26.761.340$ OR MH:C26.803.063$ OR MH:C26.874.400$
#2 MH:Artroscopia OR Artroscopia OR ARTHROSCOPY OR (Procedimentos Cirúrgicos Artroscópicos) OR MH:E01.370.388.250.070$ OR MH:E04.502.250.070$ OR MH:E04.555.113$
#3 (((single row) AND (double row)) OR ((Reparo fileira simples) AND (Reparo fileira dupla)) OR ((Sutura fileira simples) AND (Sutura fileira dupla)))
#4 #1 AND #2 AND #3 = 0
